# Editorial: Digital technology in the management and prevention of diabetes, volume III

**DOI:** 10.3389/fendo.2026.1933440

**Published:** 2026-07-24

**Authors:** Pushpita Nag, Yun Shen, Xiantong Zou, Gang Hu

**Affiliations:** 1 Pennington Biomedical Research Center, Baton Rouge, LA, United States; 2 Department of Endocrinology, Peking University People’s Hospital, Beijing, China

**Keywords:** clinical decision support, continuous glucose monitoring, diabetes mellitus, digital health, implementation science, machine learning, telemedicine

Diabetes care is increasingly shaped by the ability to convert routinely generated data into timely, individualized action. Digital technology now extends well beyond glucose meters and electronic records: it encompasses machine-learning models for risk stratification, continuous monitoring and online decision support, telehealth-enabled treatment adjustment, and interactive tools addressing the psychological work of living with diabetes. The 13 articles in this Research Topic capture that breadth across the disease continuum, from prediabetes and pediatric obesity to established diabetes, complications, comorbidity, acute illness, and long-term care. Taken together, they also sharpen a central question for the field: not simply whether a digital tool can predict an outcome, but whether it can do so transparently, equitably, and usefully within real clinical systems.

Prediction and prevention form the largest strand of the Research Topic. Yan et al. used a 5-year cohort to model reversion from prediabetes to normoglycemia, showing how machine learning may identify a window in which targeted intervention can alter disease trajectory. Moving earlier in the life course, Yang et al. developed an internally validated model for type 2 diabetes risk among children with obesity, with a support vector machine showing strong discrimination and identifying a compact set of metabolic and thyroid-related predictors. Bao et al. addressed a phenotype that may be overlooked by obesity-centered screening—lean non-alcoholic fatty liver disease in people with type 2 diabetes—and combined random forest modeling with SHAP explanations to identify influential clinical variables. These studies illustrate the appeal of using familiar measurements to detect heterogeneous risk before overt deterioration. At the same time, their predominantly retrospective or internally validated designs make external, prospective evaluation essential before clinical thresholds are adopted.

Several contributions extend prediction to complications and survival. Yan et al. combined routine laboratory indicators, ensemble learning, and Bayesian optimization to classify major diabetic complications while exploring the trade-off between predictive performance and testing cost. Liu et al. modeled nephropathy, retinopathy, peripheral neuropathy, and diabetic foot separately, revealing both shared and complication-specific risk profiles. Vershinina et al. applied survival learning and SHAP explanations to long-term all-cause mortality in type 2 diabetes, producing individualized interpretations from ten key features. Beyond conventional outpatient settings, Ge et al. found that postoperative glycemic variability was associated with mortality after craniotomy and incorporated variability measures into an interpretable random survival forest model. Huang et al. further positioned diabetes within a broader inflammation–metabolism–liver framework for severe inflammatory bowel disease, demonstrating how digital risk modeling can expose clinically meaningful interactions between diabetes and comorbidity. Collectively, these articles move the field away from a single glucose-centric endpoint toward multidimensional forecasts of organ-specific, perioperative, and long-term outcomes.

A second strand brings prediction closer to immediate treatment decisions. Liu et al. developed models for achieving time in range during short-term intensive insulin therapy, translating real-world inpatient data into a nomogram for individualized glycemic management. In older adults, Liu et al. combined continuous glucose-monitoring data with clinically accessible features to predict nocturnal hypoglycemia and deployed the model as an online calculator. This progression—from retrospective association to a tool available at the point of decision—is important, but deployment should be viewed as the beginning rather than the end of evaluation. Prospective impact studies must establish whether alerts change clinician or patient behavior, reduce hypoglycemia without increasing hyperglycemia or burden, remain calibrated as populations and treatment patterns change, and integrate safely with existing workflows.

The Research Topic also shows that digital diabetes care cannot be reduced to algorithmic accuracy. In a randomized trial, Lian et al. found that Bluetooth-enabled and traditional glucometers produced similar glycemic improvement when both were embedded in a telehealth insulin-titration program, although the Bluetooth group had fewer emergency department visits [11]. The finding is instructive: connectivity alone may not outperform a well-designed care pathway, while benefits may emerge in utilization or safety rather than glycated hemoglobin. Martín-Ávila et al. broadened the outcome set further through emoTICare, an artificial intelligence-supported serious game for adolescents with type 1 diabetes. Their pilot study reported reduced perceived illness threat and improved emotional expression, emphasizing that psychological adjustment, communication, and quality of life are legitimate targets of diabetes technology.

Implementation evidence completes the picture. Through interviews with healthcare professionals in Hong Kong primary care, Wong et al. identified the interacting policy, organizational, technical, workforce, and patient-level conditions that influence whether telemedicine is sustained. Staffing pressure, workflow complexity, digital literacy, identity verification, payment processes, and incomplete end-to-end services can all neutralize an otherwise promising intervention. Conversely, aligned financing, reliable infrastructure, training, technical support, and continued adaptation can turn a time-limited project into routine care. This contribution supplies an important counterweight to model-development studies: clinical value is co-produced by the tool, its users, and the system in which it is embedded.

**Figure 1:**
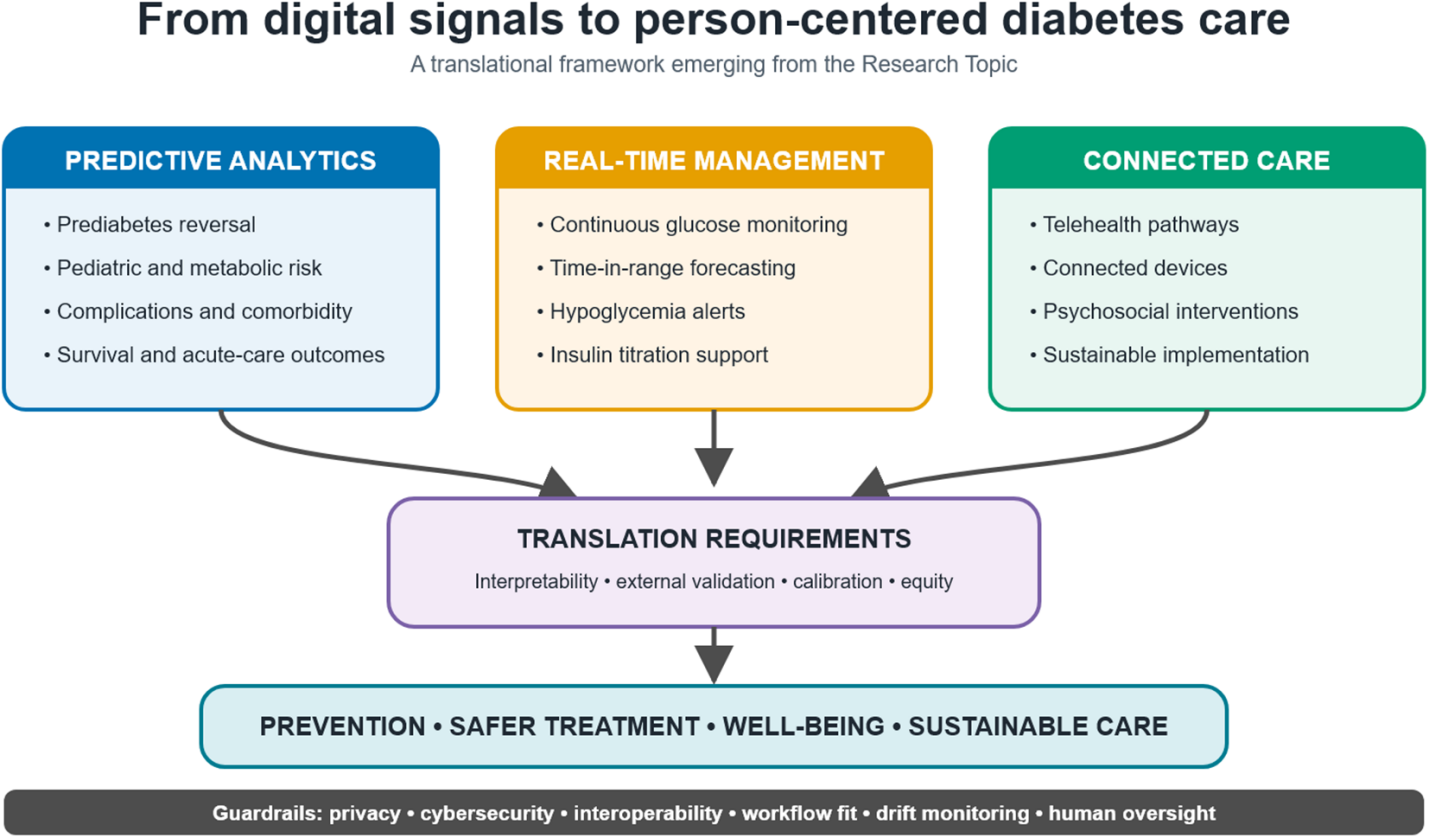
From digital signals to person-centered diabetes care. The Research Topic links three complementary domains—predictive analytics, real-time management, and connected care. Translation into meaningful outcomes requires interpretable and calibrated tools, external validation, equitable access, and governance spanning privacy, interoperability, workflow integration, model-drift monitoring, and human oversight.

Across the Research Topic, several priorities emerge (**Figure 1**). First, interpretability is becoming a design expectation, as reflected by the frequent use of SHAP and parsimonious feature sets; however, an explanation of model behavior is not equivalent to causal understanding or clinical trust. Second, transportability remains a major challenge. Multicenter external validation, subgroup assessment, calibration reporting, and transparent handling of missingness and class imbalance are necessary to determine whether performance persists across geography, age, sex, ethnicity, socioeconomic position, devices, and care settings. Third, evaluation should move beyond discrimination metrics toward clinical utility, patient-reported outcomes, workload, cost-effectiveness, safety, and equity. Finally, governance must keep pace with deployment through privacy protection, cybersecurity, auditability, interoperability, model-drift surveillance, and clear accountability when automated recommendations are wrong or unavailable.

The contributions to this volume suggest that the most consequential digital innovation in diabetes will not be a single superior algorithm or device. It will be a learning care ecosystem in which interoperable data support prevention, risk recognition, treatment adjustment, and psychosocial care while preserving meaningful human oversight. Achieving that goal will require prospective trials, implementation science, participatory design with patients and professionals, and sustained attention to people who face limited connectivity or digital literacy. We thank all authors, reviewers, and editorial staff whose work made this Research Topic possible. Their contributions show a field moving from technical possibility toward clinically grounded, person-centered digital diabetes care.

